# Requirement of direct contact between chondrocytes and macrophages for the maturation of regenerative cartilage

**DOI:** 10.1038/s41598-021-01437-6

**Published:** 2021-11-18

**Authors:** Kengo Kanda, Yukiyo Asawa, Ryoko Inaki, Yuko Fujihara, Kazuto Hoshi, Atsuhiko Hikita

**Affiliations:** 1 Department of Sensory and Motor System Medicine, Graduate School of Medicine, The University of Tokyo, Tokyo, Japan; 2 Department of Tissue Engineering, The University of Tokyo Hospital, Tokyo, Japan; 3 Department of Tissue Stem Cell and Dental Life Science, Graduate School of Medicine, The University of Tokyo, Tokyo, Japan; 4 Department of Oral-Maxillofacial Surgery, Dentistry and Orthodontics, The University of Tokyo Hospital, Tokyo, Japan

**Keywords:** Regenerative medicine, Tissue engineering

## Abstract

Regenerative cartilage prepared from cultured chondrocytes is generally immature in vitro and matures after transplantation. Although many factors, including host cells and humoral factors, have been shown to affect cartilage maturation in vivo, the requirement of direct cell–cell contact between host and donor cells remains to be verified. In this study, we examined the host cells that promote cartilage maturation via cell–cell contact. Based on analysis of the transplanted chondrocytes, we examined the contribution of endothelial cells and macrophages. Using a semiclosed device that is permeable to tissue fluids while blocking host cells, we selectively transplanted chondrocytes and HUVECs or untreated/M1-polarized/M2-polarized RAW264.7 cells. As a result, untreated RAW264.7 cells induced cartilage regeneration. Furthermore, an in vitro coculture assay indicated communication between chondrocytes and RAW264.7 cells mediated by RNA, suggesting the involvement of extracellular vesicles in this process. These findings provide insights for establishing a method of in vitro cartilage regeneration.

## Introduction

Cartilage tissue exists in various parts of the body, such as the nose, auricles and joints, maintaining a morphological outline and enabling the activities of daily life. Cartilage is affected by congenital diseases such as microtia and nasal deformity due to cleft lip and palate, injury, and malignant tumours. Because cartilage has a poor ability to regenerate due to the lack of blood vessels, cartilage transplantation is a major clinical option to reconstruct damaged, deformed or hypoplastic cartilage^1^. Cartilage autografts are often used for cartilage reconstruction, and healthy donor sites are largely damaged^2–4^. To solve the problem of donor site morbidity, regenerative methods of cartilage reconstruction have been developed.

In 1987, Brittberg et al*.*^5^ proposed a technique known as autologous chondrocyte implantation (ACI), in which chondrocytes are harvested from non-weight bearing sites with minimum invasion, expanded and transplanted to the damaged site. Another group reported a two-stage transplantation method, in which cultured chondrocytes were injected subcutaneously into the patient's lower abdomen for maturation, and the matured cartilage tissue was harvested and used for nasal reconstruction^6^. Recently, our group developed implant-type regenerative cartilage for the reconstruction of nose deformation caused by a cleft lip and palate^7^. Auricular chondrocytes from a small fragment of autologous ear cartilage were expanded and transplanted with a poly-l-lactic acid scaffold.

In these regenerative methods utilizing chondrocytes, the cells are isolated from cartilage and expanded in vitro. During the culture period, the cells dedifferentiate and lose their ability to produce cartilage matrix^8^. Because cartilage tissue mainly matures after transplantation, the extent of cartilage regeneration could be affected by the local environment and health of the patients, and the effectiveness of the treatment becomes rather uncertain. Yamashita et al*.*^9^ developed a method to induce cartilage regeneration in vitro using human induced pluripotent stem (iPS) cells, but long-term safety and the very high cost hinder the clinical use of iPS cells^10^. Therefore, a method to obtain mature cartilage tissue from chondrocytes in vitro is worth developing. To this end, factors that induce chondrogenesis in vivo should be identified.

Several factors, such as host cells and growth factors, are involved in the redifferentiation of transplanted chondrocytes and the maturation of cartilage tissue^11–16^. Previous papers have reported the interaction between transplanted chondrocytes and host cells in vivo. Takebe et al*.*^17^ first reported the supportive role of endothelial cells in cartilage regeneration from human ear–derived cartilage progenitor cells (CPCs). The researchers indicated that the early interactions of CPCs with endothelial cells triggered the initial expansion of CPCs and promoted the self-aggregation of a 3D group of progenitors without a scaffold or exogenous factors. Fujihara et al*.*^18,19^ showed interactions between transplanted chondrocytes and host macrophages. The researchers indicated that chondrocytes express macrophage migration inhibitory factor (MIF) and fas ligand (FasL), presumably in response to cytokines or humoral factors produced by macrophages^18^. MIF is expressed by chondrocytes and promotes the maturation of chondrocytes at an earlier stage, while it impairs the maturation of tissue-engineered cartilage in the long term^19^. The authors also showed that depletion of M1 macrophages in the early phase improved chondrogenesis in transplanted regenerative cartilage^20^. Yamawaki et al*.*^21^ examined transplanted chondrocytes on coverslips by electron microscopy and indicated that chondrocytes and macrophages were in contact in the early phase of cartilage regeneration.

While these studies suggested direct cell–cell contact during chondrogenesis in vivo, humoral factors could also affect cartilage regeneration. When cultured with chondrocytes, mesenchymal stem cells (MSCs) improved chondrogenesis and suppressed chondrocyte hypertrophy and mineralization^22–25^. Some reports have indicated that the trophic and paracrine effects of MSCs are more important for enhancing the cartilage-forming capacity of chondrocytes than the chondrogenic differentiation of MSCs themselves^25–28^. Indeed, exosomes harvested from MSC culture media can also induce cartilage regeneration^29^.

In this report, we examined the requirement of direct contact between host and donor cells for cartilage regeneration in vivo. We used a semiclosed device that could prevent contact with host cells, while humoral factors, including proteins and ions, could reach chondrocytes. Chondrocytes were seeded on the inner side of this device and transplanted to examine cartilage regeneration in the absence of direct contact with host cells. We also examined the cells required for cartilage regeneration by selectively adding endothelial cells or macrophages to this device before transplantation.

## Results

### In vivo transplantation of the device with chondrocytes on the outer bottom or inner bottom

**Figure 1:**
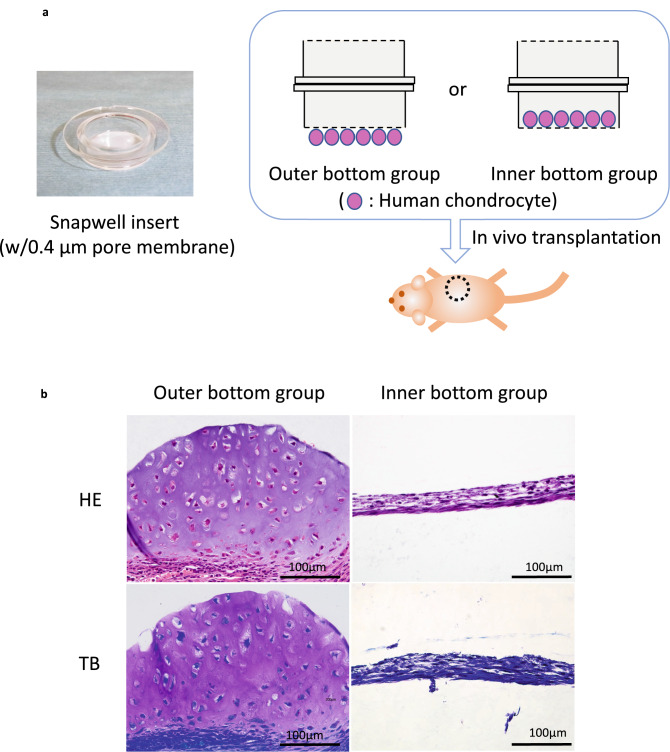
(**a**) Snapwell insert and schematic of the device (inner and outer bottom groups). (**b**) Histological images of the transplanted chondrocytes at 4 weeks posttransplantation(HE: haematoxylin & eosin staining, TB: toluidine blue staining). Cartilage matrix was formed only in the outer bottom group.

To confirm the involvement of host cells in the chondrogenesis of transplanted chondrocytes, devices were prepared using 2 membrane inserts (Fig. 1a). Chondrocytes were seeded on the inner side of the membrane (inner bottom group) to prevent direct contact with host cells, while soluble factors were allowed to pass through the membrane. For positive controls, chondrocytes were seeded on the outer side of the membrane (outer bottom group). At 4 weeks posttransplantation, cartilage matrix with lacunas was detected in the outer bottom group (Fig. 1b). In contrast, no cartilage matrix was formed in the inner bottom group, suggesting the requirement of direct contact between host cells and donor chondrocytes for chondrogenesis (Fig. 1b).

### Host cells involved in the early stage of cartilage matrix formation

**Figure 2:**
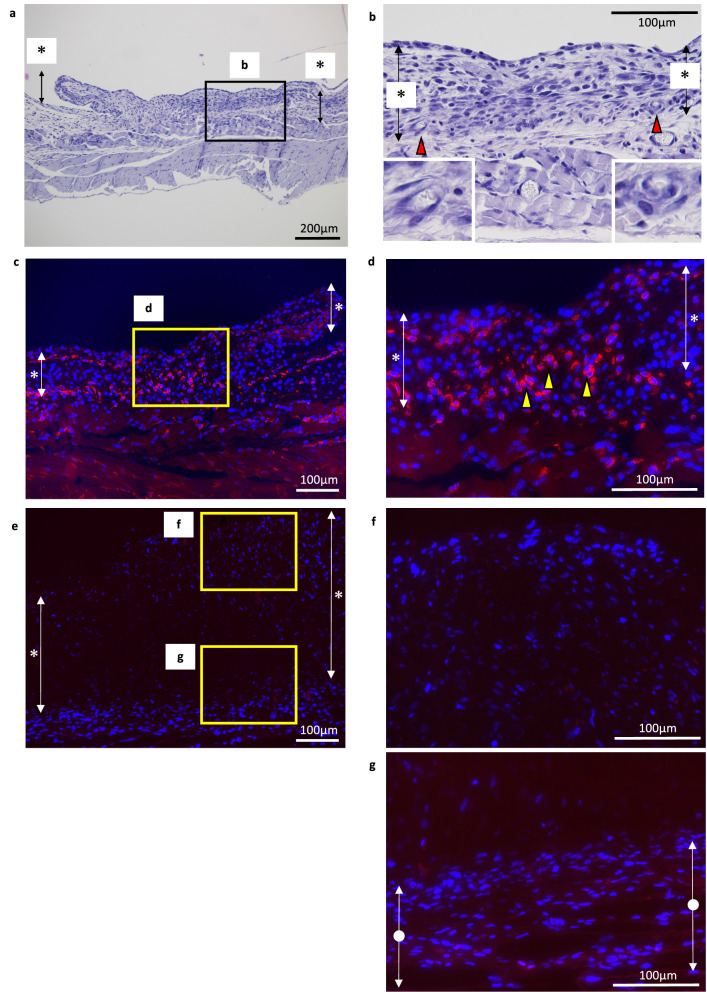

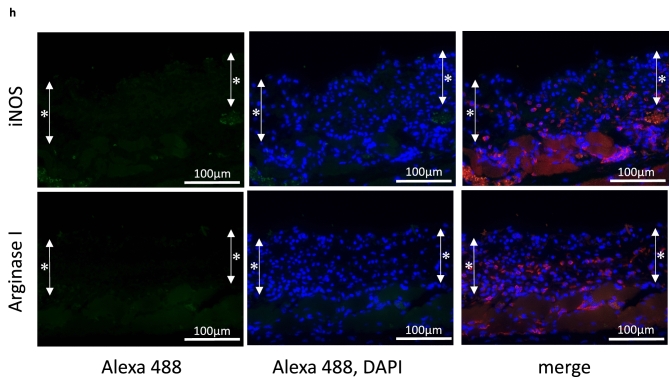
(**a**–**d**) Histological images of the outer bottom group at 1 week posttransplantation. Asterisks indicate the transplanted chondrocyte layer. (**a**) HE staining. (**b**) An enlarged view of the area indicated by the black rectangle in (**a**). Capillaries are indicated by red arrowheads with enlarged views at the corners. (**c**,**d**) An immunofluorescent image of the same sample as in (**a**). Asterisks indicate the transplanted chondrocyte layer. Alexa 594: F4/80, DAPI: nucleus. (**d**) An enlarged view of the area indicated by the yellow rectangle in (**c**). F4/80-positive cells (yellow triangle) were observed among the transplanted chondrocytes. (**e**–**g**) Immunofluorescent images of the outer bottom group sample at 2 weeks posttransplantation. Alexa 594: F4/80, DAPI: nucleus. Asterisks indicate the transplanted chondrocyte layer, and white circles indicate the host cell layer. (**f**,**g**) Enlarged views of areas indicated by yellow rectangles in (**e**). F4/80-positive cells were rarely observed in either the transplanted chondrocyte layer or the host layer. (**h**) Immunofluorescent images of the outer bottom group sample at 1 week posttransplantation. Asterisks indicate the transplanted chondrocyte layer. (upper row) Alexa 488: iNOS, Alexa 594: F4/80, DAPI: nucleus. (lower row) Alexa 488: Arginase I, Alexa 594: F4/80, DAPI: nucleus.

To identify the host cells that are involved in cartilage matrix formation, the cells that were present 1 week after transplantation in the outer bottom group were examined histologically. Some capillaries surrounded by flat cells and containing blood cells were observed in the bottom layer of transplanted chondrocytes (Fig. 2a,b). F4/80-positive cells were also detected in the transplanted chondrocyte layer (Fig. 2c,d). These findings suggest that endothelial cells or macrophages are involved in cartilage matrix formation by transplanted chondrocytes.

### F4/80-positive cells at 2 weeks posttransplantation

In a previous study, macrophages were observed around transplanted chondrocytes at early time points, and these cells disappeared at later stages^21^. To examine the involvement of macrophages in chondrogenesis at later time points, at 2 weeks after transplantation, histological sections from the outer bottom group were stained with the F4/80 antibody. In contrast to samples at 1 week posttransplantation (Fig. 2c,d), few F4/80-positive cells were observed in or around the transplanted chondrocyte layer (Fig. 2e–g). This result indicates an early contribution of macrophages to cartilage matrix formation.

### Characterization of F4/80-positive cells at 1 week posttransplantation

To investigate the character of the F4/80-positive cells at 1 week posttransplantation, we doublestained the samples at 1 week posttransplantation with the iNOS antibody as M1 marker or the Arginase I antibody as M2 marker together with the F4/80 antibody. Any F4/80-positive cells in the transplanted chondrocyte layer were not detected to be positive for iNOS or Arginase I in three fields of view each (representative image: Fig. 2h, positive control: Supplementary Fig. S1), indicating unpolarized macrophages contribute to the cartilage maturation.

### Effects of HUVECs on cartilage matrix formation

**Figure 3:**
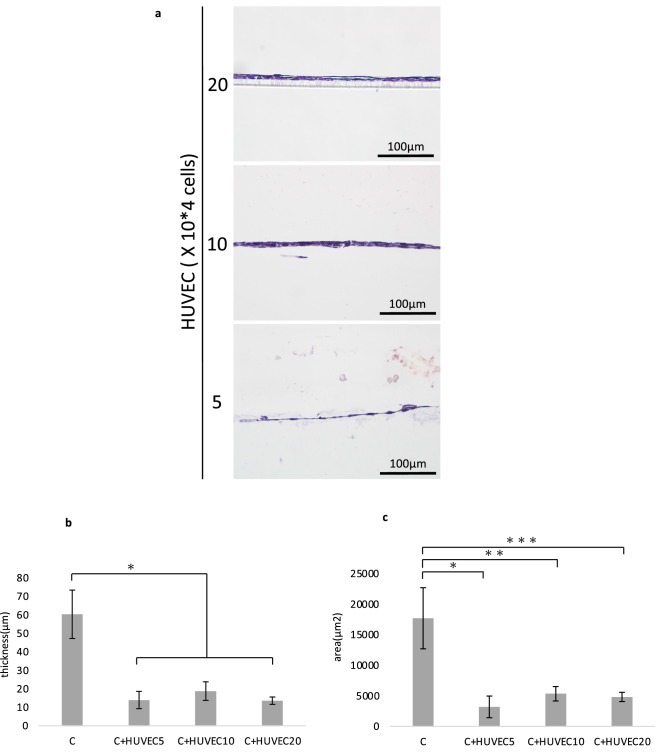
(**a**) Histological images of the chondrocyte + HUVEC inner bottom group (4 weeks posttransplantation, HE staining). Cartilage matrix was not observed in any group. (**b**,**c**) Statistical analysis of the thickness and area of chondrocytes alone and chondrocytes + HUVECs in the bottom group. The data are shown as the means ± S.D. n = 3. (**b**) Comparison of the thickness. *p < 0.001. (**c**) Comparison of the area. *p = 0.00105, **p = 0.00244, ***p = 0.00190.

To examine the effect of endothelial cells on cartilage matrix formation, different numbers of HUVECs were added to the chondrocytes in the inner bottom group just before transplantation. Histological analysis indicated that at 4 weeks after transplantation, no cartilage matrix was formed in the presence of any number of HUVECs (Fig. 3a). Furthermore, the tissue size seemed to be less than that in the device containing only chondrocytes (Fig. 1b). We compared the thickness and the area of the tissues from the device containing chondrocytes alone and chondrocytes plus different numbers of HUVECs (Fig. 3b,c). The thickness and area of the tissues in the device containing chondrocytes and different numbers of HUVECs were significantly smaller than those of the tissues in the device containing only chondrocytes (thickness: p < 0.001, area: p < 0.01).

### Pretreatment of RAW264.7 cells before transplantation

**Figure 4:**
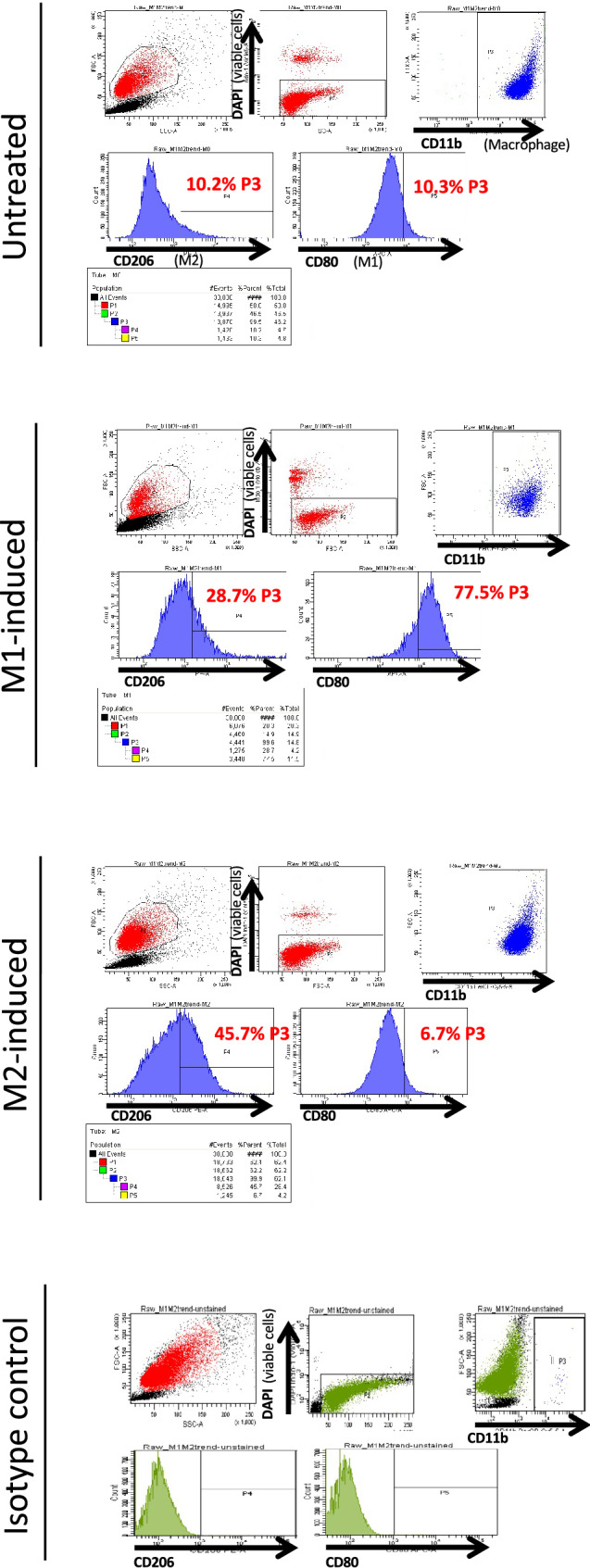
Confirmation of RAW264.7 cell polarization by flow cytometry.

Macrophages can be polarized into several subgroups. Generally, M1 macrophages are inflammatory, while M2 macrophages suppress inflammation and induce tissue repair^30,31^. To examine the effects of different macrophage subtypes on chondrogenesis, 3 types of RAW264.7 cells were prepared and then added to the inner bottom. We compared populations of cells in the region where top 10% of untreated RAW264.7 cells positive for CD206 and CD80 were included (defined as CD206^high^ and CD80^high^, respectively) by flow cytometry (Fig. 4). The results showed that 77.5% of M1-induced RAW264.7 cells were CD80^high^ and 45.7% of M2-induced RAW264.7 cells were CD206^high^, confirming successful induction of RAW264.7 cells into each subtype.

### Effects of RAW264.7 cells on cartilage matrix formation

**Figure 5:**
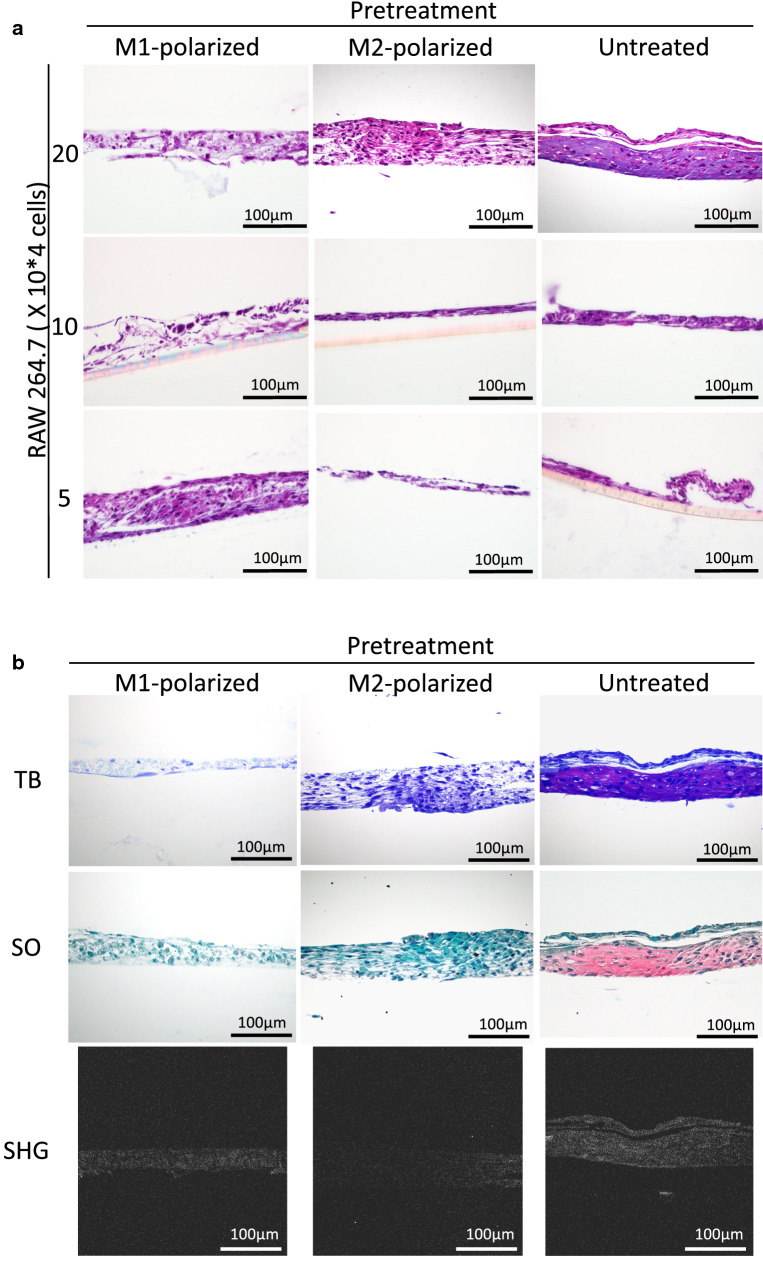

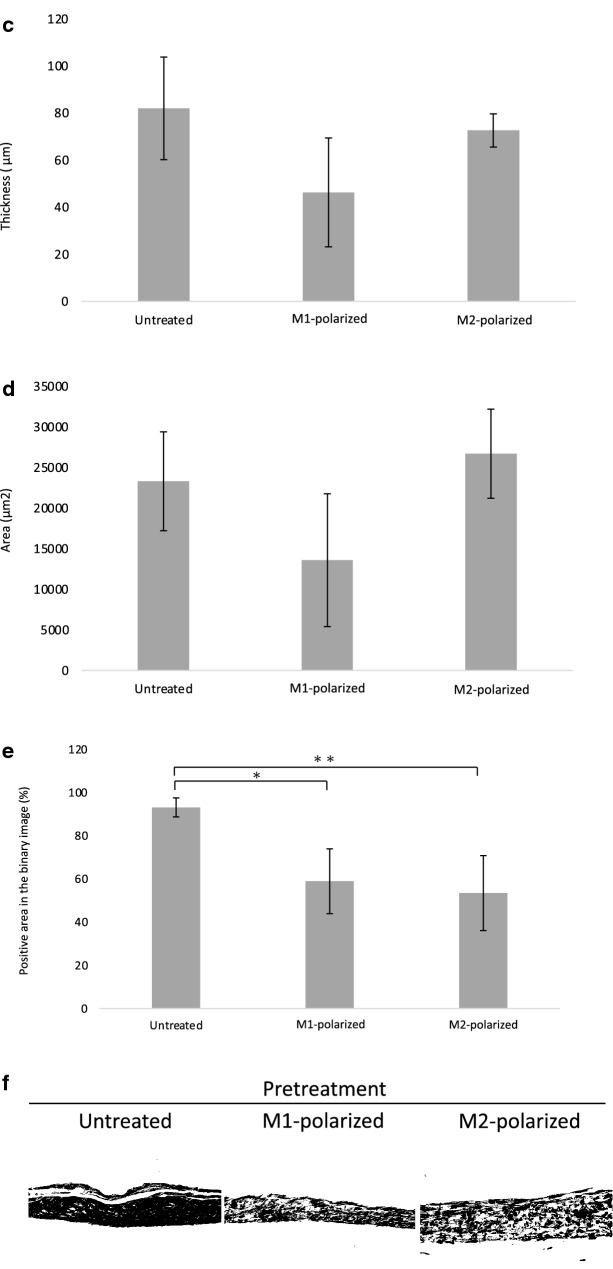
(**a**) Histological images of the chondrocyte + RAW264.7 inner bottom group (4 weeks posttransplantation, HE staining). Cartilage-like tissue was observed in the group that was transplanted with 20 × 10^4^ untreated RAW264.7 cells. (**b**) Cartilage matrix staining and SHG images of chondrocytes + RAW264.7 cells in the inner bottom group (20 × 10^4^ RAW264.7 cells, 4 weeks posttransplantation, TB: toluidine blue staining, SO: safranin-O staining). Cartilage matrix was observed in the untreated group. (**c**–**e**) The thickness, area and percentage of the positive area in the binarized image of the chondrocyte + RAW264.7 inner bottom group (20 × 10^4^ RAW264.7 cells, 4 weeks posttransplantation). The data are shown as the means ± S.D. n = 3. (**c**) Comparison of the thickness. (**d**) Comparison of the area. (**e**) Comparison of the percentage of the binarized positive area. *p = 0.0477, **p = 0.0264. (**f**) Binarized images of the chondrocyte + RAW264.7 inner bottom group (4 weeks posttransplantation, HE staining).

Different numbers of RAW264.7 cells (untreated, M1-polarized, M2-polarized) were added to chondrocytes in the inner bottom group just before transplantation. Cartilage matrix-like tissue was detected in the group that contained 20 × 10^4^ untreated RAW264.7 cells (Fig. 5a). In some other groups, cells seemed to have proliferated, but cartilage-like tissues with lacunae were not detected.

To further confirm chondrogenesis, histological sections were examined with toluidine blue staining and safranin-O staining, and second harmonic generation (SHG) images were acquired. Metachromasia was shown by toluidine blue staining, reddishness was shown by safranin-O staining, and a stronger SHG signal indicated cartilage matrix formation in the group that was transplanted with 20 × 10^4^ untreated RAW264.7 cells (Fig. 5b). To confirm chondrogenesis, we also compared the thickness, area and positive area of the binarized image (Fig. 5c–f). No significant difference was observed in either the thickness or the area [thickness: p = 0.0761, area: p = 0.118 (one-way ANOVA)]. However, the positive area of the binarized image was significantly increased in the group transplanted with untreated cells compared to those transplanted with M1- or M2-polarized cells (untreated vs. M1: p = 0.0263; untreated vs. M2: p = 0.0477). The increased tissue density indicated the generation of cartilage matrix.

### Communication between chondrocyte and RAW264.7 cells

**Figure 6:**
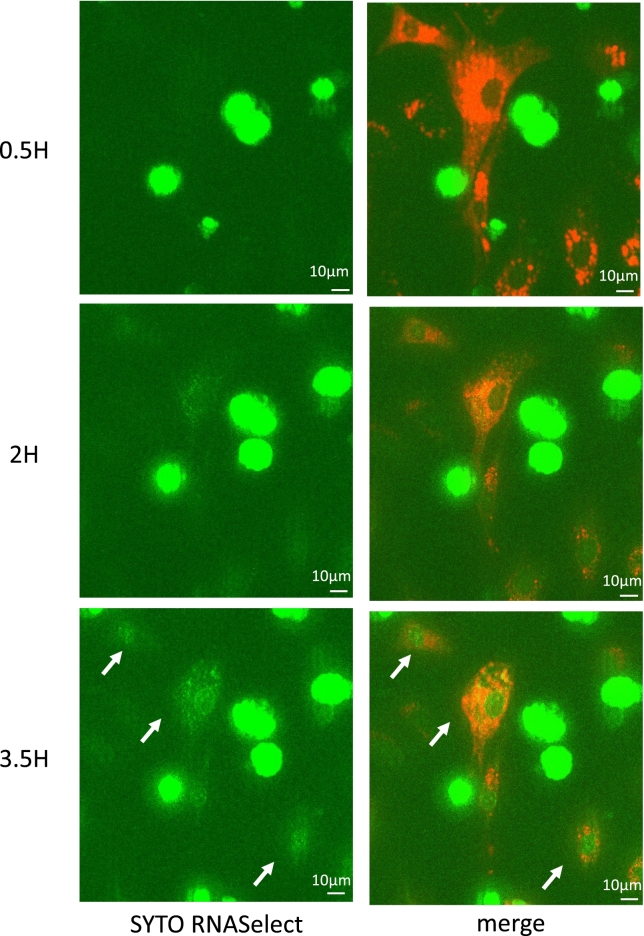
In vitro communication between chondrocytes and RAW264.7 cells. Green spots (indicating RNA from RAW264.7 cells) appeared gradually in chondrocytes. Red (CM-DiI): chondrocyte, green (SYTO RNASelect): RNA from RAW264.7 cells.

To confirm the occurrence of substance transfer from RAW264.7 cells to chondrocytes, fluorescent dye transfer was examined in the coculture system. After the adhesion of CM-DiI-stained chondrocytes to the culture slide, SYTO RNASelect-stained RAW264.7 cells were added to the same slide. At 3.5 h, SYTO RNASelect-positive points gradually increased in cocultured chondrocytes (Fig. 6, white arrows), indicating the occurrence of some communication between chondrocytes and RAW264.7 cells.

**Figure 7:**
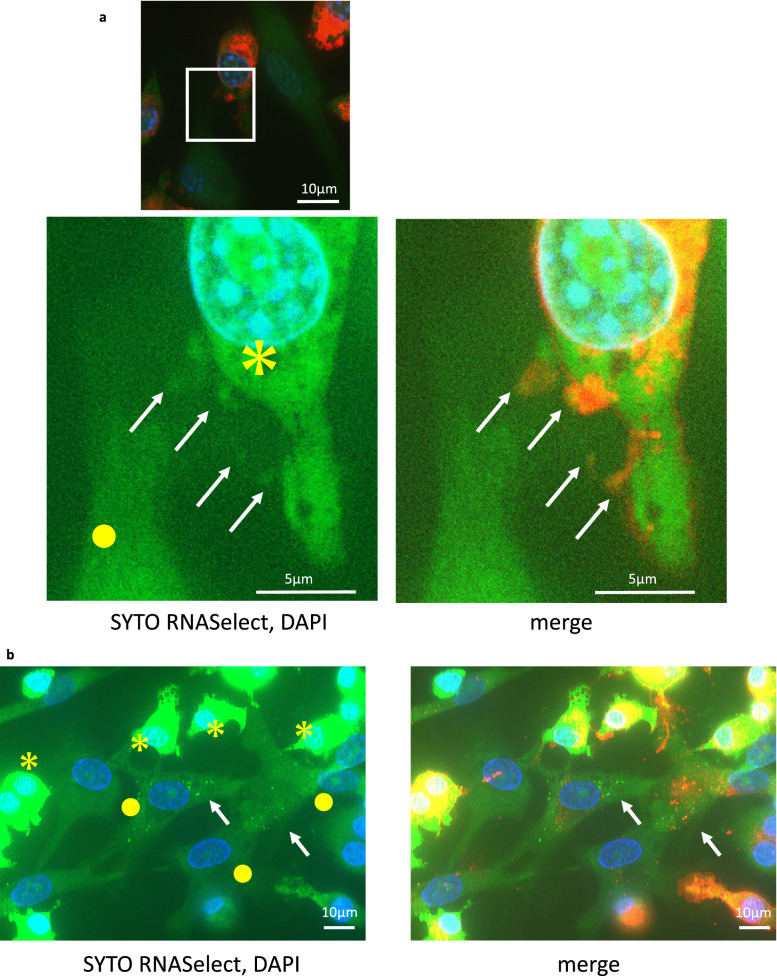

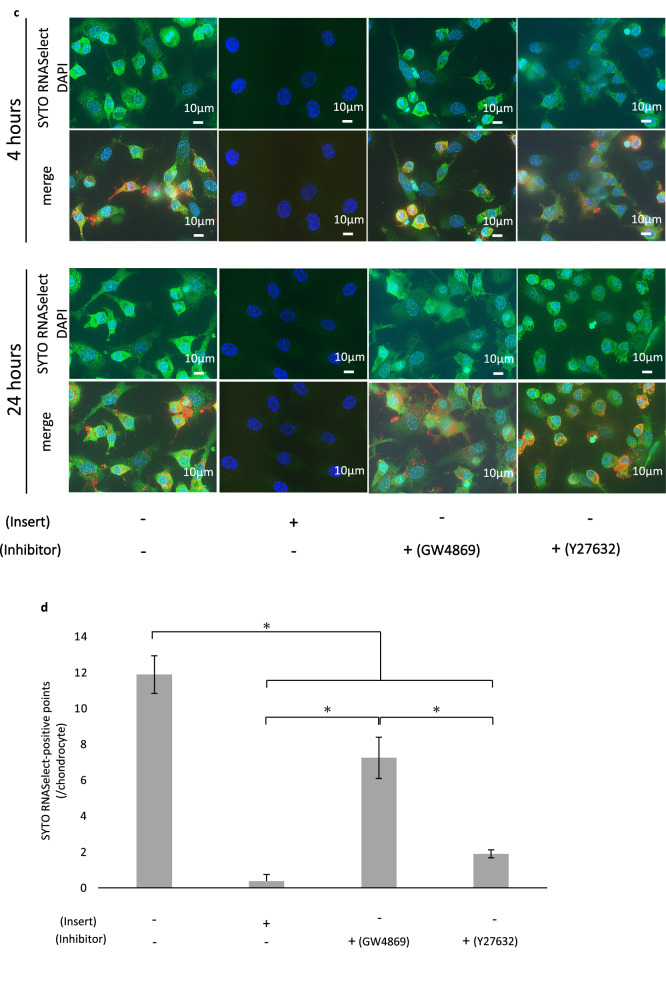
Tracking the cell membrane components during in vitro communication between chondrocytes and RAW264.7 cells. Red (CM-DiI): cell membrane of RAW264.7 cells, green (SYTO RNASelect): RNA from RAW264.7 cells. (**a**,**b**) Yellow asterisks indicate RAW264.7 cells, and yellow circles indicate chondrocytes. (**a**) Fluorescence images of cells after 4 h of coculture. SYTO RNASelect-positive points were outlined by CM-DiI (white arrow). (**b**) Fluorescence images of cells after 24 h of coculture. Chondrocytes contained RNA from RAW264.7 cells (white arrow). (**c**) Fluorescence images of four groups after 4 and 24 h of coculture. (**d**) SYTO RNASelect-positive points count per chondrocyte. The data are shown as the means ± S.D. *p < 0.001. n = 3.

Additionally, to examine the contribution of extracellular vesicles (EVs) to communication, RAW264.7 cells were stained with both CM-DiI and SYTO RNASelect and cocultured with unstained chondrocytes. At 4 h, SYTO RNASelect-positive points were outlined by CM-DiI (Fig. 7a). This result indicated that some EVs contribute to communication. At 24 h, chondrocytes contained some cytoplasmic SYTO RNASelect-positive points (Fig. 7b), indicating the accumulation of the RNA transferred from RAW264.7 cells.

To further examine the mode of communication, we prepared 3 coculture groups of unstained chondrocytes and RAW264.7 cells labelled with both CM-DiI and SYTO RNASelect: (1) direct coculture, (2) indirect coculture with inserts, and (3) direct coculture with EV inhibitors (Fig. 7c). In group (3), we used 2 inhibitors to determine which subgroup of EVs contributed to communication. GW4869 was used to inhibit exosome generation and release, and Y27632 was used to inhibit microvesicle (MV) production.

Moreover, we compared the number of SYTO RNASelect-positive points within chondrocytes (Fig. 7d). The significant difference between groups (1) and (2) indicated that even though the membrane pore size (0.4 µm) was much larger than EVs, the distance between chondrocytes and RAW264.7 cells imposed by the Snapwell insert membrane inhibited communication [(1) vs. (2): p < 0.001]. This result can explain the lack of maturation in the inner bottom group in the in vivo experiment (Fig. 1b). Y27632 most effectively suppressed the uptake of SYTO RNASelect [(1) vs. (3) Y27632: p < 0.001; (2) vs. (3) Y27632: p = 0.0659], and GW4869 was less effective at suppressing uptake [(1) vs. (3) GW4869, (3) GW4869 vs. (3) Y27632, (2) vs. (3) GW4869: p < 0.001]. This result indicates that MVs play a large role in communication, and exosomes contribute to some degree.

## Discussion

Many factors affect transplanted cartilage regeneration, including host cells and humoral factors, such as inductive growth factors. In this report, we focused on host cells that trigger the redifferentiation of monolayer-cultured chondrocytes through direct cell–cell contact. Host cells such as endothelial cells, macrophages and MSCs have been reported to help the maturation of chondrocytes^17–29^, and endothelial cells and macrophages have been reported to directly communicate with chondrocytes. By analysing transplanted chondrocyte maturation in vivo, we confirmed the presence of endothelial cells and macrophages, in addition to transplanted chondrocytes. We hypothesized that endothelial cells or macrophages communicated with chondrocytes, combined each type of cell with chondrocytes in vivo, and examined the maturation of chondrocytes. However, other cell types such as MSCs and cells of hematopoietic lineage may also affect the chondrogenesis. To elucidate the process of cartilage maturation in vivo, these cell types should be analysed in future studies.

In contrast to the result of Takebe et al*.*^17^ HUVECs did not cause chondrocytes to mature in our model. Takebe et al*.* precultured a mixture of CPCs and HUVECs, and the mixture formed 3D vascularized condensed progenitors. They transplanted this 3D condensation in vivo and concluded that HUVECs supported chondrocyte maturation. HUVECs were added to monolayer-cultured chondrocytes just before transplantation in the present study. Our results do not contradict the supportive effects of endothelial cells on chondrogenesis in a 3D context but suggest that the host cells that induce in vivo maturation of monolayer-cultured chondrocytes are not endothelial cells. In contrast to our expectations, the addition of HUVECs to the device seemed to suppress the tissue formation. Although additional studies are needed to describe the cell population and viability posttransplantation, the doubled cell numbers compared with the transplantation of chondrocytes alone may have caused low proliferation or cell death, which led to reduced tissue size.

In the case of RAW264.7 cells, we prepared untreated, M1- and M2-polarized cells, anticipating the advantage of M2-polarized cells for cartilage regeneration^30,31^. Fujihara et al*.*^18,20^ reported that the early macrophage population is supposed to be shifted to M1, and the depletion of M1 macrophages promotes chondrogenesis. In our study, unpolarized macrophages were detected around chondrocytes seeded on the outer membrane at 1 week but not 2 weeks, and untreated RAW264.7 cells worked more effectively than M1- or M2-polarized RAW264.7 cells when transplanted with chondrocytes. This finding suggests that unpolarized macrophages trigger the very early stage of chondrocyte maturation. Our fluorescent dye transfer assay also supported this early effect by indicating very early communication between chondrocytes and RAW264.7 cells in vitro. Taken together with past studies, our results indicate that each polarized macrophage subtype plays different roles in maturation stages. Further analysis is required to clarify whether untreated RAW264.7 cells work as they are or whether these cells polarize into other states in vivo after transplantation.

The device used in this study is open to tissue fluids and closed to cells because of the membrane pore size, which means that only the cell enclosed in the device with chondrocytes can communicate directly (or in very close proximity) with chondrocytes. Our results indicate that direct communication between chondrocytes and macrophages is necessary for cartilage maturation. Yamawaki et al*.*^21^ presented TEM images of in vivo transplanted chondrocytes, in which macrophage-like cells were indicated to have cell–cell contact with chondrocytes in the first week. Between those two cells, small structures resembling EVs were observed around the contact site. These structures resemble the EVs observed around macrophages, as shown by TEM in another report^32^. EVs are thought to facilitate intercellular communication by transferring nucleic acids such as mRNAs and miRNAs, their fragments and proteins between cells to induce biological responses in recipient cells depending on the cargo^33,34^. To clarify the occurrence of communication between chondrocytes and RAW264.7 cells, we focused on RNA transfer. We observed early communication within several hours between these cells. We also observed RAW264.7 RNA outlined by the plasma membrane component of RAW264.7 cells, suggesting that EVs budded from the plasma membrane of RAW264.7 cells. This finding suggests that EVs are involved in intercellular communication between RAW264.7 cells and chondrocytes. According to the International Society of EVs, three main subgroups of EVs have been classified based on size, membrane composition, and the mechanism of formation^35^. In this study, the EV-like structure we observed could be MVs based on their sizes, and they were much smaller than the membrane pores of the Snapwell insert and could pass through the membrane. Moreover, analysis of chondrocyte uptake of RAW264.7 cell RNA indicated that MVs contribute much more to this communication than exosomes. However, further investigations are needed to examine other features of MVs, such as membrane markers and their mechanisms of formation.

In conclusion, we indicated the necessity of direct contact between transplanted chondrocytes and host macrophages for cartilage differentiation. In the early stage of maturation, unpolarized macrophages affected cartilage maturation most effectively relative to other types of macrophages. Moreover, we showed that RNA transfer between chondrocytes and macrophages occurred by EVs in vitro, which indicates some early effect of macrophages on chondrocytes. These results provide new insight for engineering regenerative cartilage in vitro*.*

## Methods

### Cell preparation

All procedures were approved by the Research Ethics Committee of the University of Tokyo Hospital (permission number 2573). Human auricular cartilage was obtained with informed consent during ear reconstruction surgeries for microtia at Nagata Microtia and Reconstructive Plastic Surgery Clinic. After the soft tissues and perichondria were removed, the auricular cartilage was cut into small pieces and digested in 0.3% collagenase for 18 h at 37 °C. After being filtered through a cell strainer (100 μm pore size, BD Falcon), the solution was centrifuged at 1500 rpm for 5 min to collect chondrocytes. Two hundred thousand cells were seeded onto 100 mm collagen type I-coated dishes (AGC Techno Glass Co., Ltd.) and cultured with Dulbecco's modified Eagle’s medium/nutrient mixture F-12 (DMEM/F12; Sigma-Aldrich Co.) supplemented with 5% human serum (Sigma-Aldrich Co.), 100 ng/mL FGF-2 (Kaken Pharmaceutical Co, Ltd.), 5 μg/mL insulin (Novo Nordisk Pharma Ltd.) and 1% penicillin/streptomycin (Sigma-Aldrich Co.) (cartilage growth medium: HFI) at 37 °C in a humidified atmosphere containing 5% CO_2_ for 10 days until the cells reached confluence. The cells were collected using trypsin–EDTA (Sigma-Aldrich Co.) and stored with CELLBANKER (Nippon Zenyaku Kogyo Co., Ltd.) at − 80 °C. The HUVEC line and RAW264.7 cell line were purchased from Lonza and ATCC, respectively. For the in vivo coculture assay, RAW264.7 cells were cultured until 70% confluent in growth medium consisting of DMEM/F12 (Sigma-Aldrich Co.) supplemented with 10% foetal bovine serum (Gibco) and 1% penicillin/streptomycin (Sigma-Aldrich Co.) and were induced to polarize. For M1 or M2 polarization, LPS (50 ng/mL; Wako) and GM-CSF (20 ng/mL; Fujifilm) or IL-4 (20 ng/mL; Fujifilm) and M-CSF (50 ng/mL; Wako), respectively, were added to the growth medium, and the cells were cultured for 24 h at 37 °C in a humidified atmosphere containing 5% CO2. For the untreated group, cells were cultured in growth medium for 24 h.

To confirm the polarization, the suspension of RAW264.7 cells was diluted to 1 × 10^7^ cells/ml with FACS staining buffer (BioLegend, San Diego, USA), and the following antibodies were added: anti-mouse CD11b PerCP-Cyanine 5.5 (0.25 μg/test; 45–0112-82, eBioscience) as a macrophage marker, anti-mouse CD80 APC (0.06 μg/test; 17–0801-82, eBioscience) as an M1 macrophage marker, and anti-mouse 206 PE (0.25 μg/test; 12–2061-82, eBioscience) as an M2 macrophage marker. The cells were incubated with antibodies for 20 min at 4 °C in the dark, after which they were washed and stained with DAPI (BD Pharmingen, USA) to assess viability. Fluorescence was analysed with a Becton Dickinson LSR Fortessa cell analyser using BD Diva software. Compensation measurements were performed for single stains using compensation beads (eBiosciences). The samples stained with IgG isotype antibodies [rat IgG2b K isotype control PerCP-cyanine 5.5 (0.125 µg/test; 45-4031-80, eBioscience), Armenian hamster IgG isotype control APC (0.03 µg/test; 17-4888-82, eBioscience), and rat IgG2b K isotype control PE (0.06 µg/test; 12-4031-82, eBioscience)] were used as the negative controls.

All methods were performed in accordance with the relevant guidelines and regulations.

### Preparation of the closed device and transplantation

Frozen stocks of P0 chondrocytes were thawed, cultured with HFI, and passaged using trypsin–EDTA. Fifty thousand P2 chondrocytes were seeded on the inner side or the outer side of a 12 mm Snapwell insert with a 0.4 µm pore polyester membrane (Corning) and cultured in HFI until confluent, at which time the cell number reached approximately 2 × 10^5^. To prepare a closed environment for the seeded chondrocytes, the membrane of the Snapwell insert seeded with cells was adhered to another insert without cells using the instant adhesive Aron Alpha. For coculture assays, 20 × 10^4^, 10 × 10^4^, or 5 × 10^4^ HUVECs or RAW264.7 cells were added before adhesion of the 2 inserts. The device was transplanted into the back subdermal space of 6-week-old male Balb/c-nu/nu mice (CLEA Japan, Inc.) with the cell-seeded membrane facing the muscle side. One device was transplanted in each mouse (n = 3). At certain time points, the device and the tissue directly below the device were collected and analysed histologically. Protocols for animal experiments were approved by the Institutional Animal Care and Use Committee of the University of Tokyo (#P19-073). All methods were performed in accordance with the relevant guidelines and regulations. The authors complied with the ARRIVE guidelines.

### Histological analysis

Each sample was fixed with 4% paraformaldehyde overnight, and 5 μm-thick paraffin-embedded sections were prepared. The sections were generally stained with haematoxylin and eosin. Toluidine blue staining and safranine-O staining were also performed to examine the cartilage matrix. For immunofluorescence analysis with the F4/80 antibody (ab6640, Abcam), after antigen retrieval and blocking with 10% BSA in PBS, the sections were incubated with primary antibody (1:100 dilution) for 1 h at 37 °C and fluorescent secondary antibodies (1:200 dilution) for 30 min at room temperature. The coverslips were subsequently mounted using mounting medium containing DAPI. For the double staining with the F4/80 and the iNOS antibody (PA3-030A, Thermo Fisher Scientific), after antigen retrieval and blocking with 10% BSA in PBS-T, the sections were incubated with primary antibody (F4/80; 1:100 dilution, iNOS; 1:250 dilution) over night at 4 °C and fluorescent secondary antibodies (1:500 dilution) for 30 min at room temperature. For the double staining with the F4/80 and the Arginase I antibody (sc-20150, Santa Cruz Biotechnology), after antigen retrieval and blocking with 10% BSA in PBS, the sections were incubated with primary antibody (F4/80; 1:100 dilution, Arginase I; 1:50 dilution) for 1 h at 37 °C and fluorescent secondary antibodies (1:200 dilution) for 30 min at room temperature. As positive controls for iNOS and Arginase I staining, histological sections of mouse liver were stained by the same procedures.

For SHG images, we used a multiphoton confocal microscopy system (A1R + MP, Nikon), an excitation laser (Mai Tai eHP, wavelengths: 690–1040 nm; repetition rate: 80 MHz; pulse width: 70 fs, Spectra-Physics, Tokyo, Japan) and a water-immersion objective lens (CFI75 Apo 25 × W MP, numerical aperture: 1.1, Nikon). The excitation wavelength was 950 nm. The thickness and area of the HE samples were measured by cellSense (Olympus). In addition, HE samples were binarized by the same parameter by ImageJ^36^, and we compared the positive area to the entire area before binarization was performed.

### Coculture and fluorescent dye transfer assay

P2 chondrocytes were trypsinized, suspended in 25 μM CM-DiI (V22888, Invitrogen) diluted in DMEM/F12 supplemented with 1% penicillin/streptomycin, and incubated for 30 min at 37 °C. The cells were washed twice and suspended in the same medium. RAW264.7 cells were labelled with 5 μM SYTO RNASelect (S32703, Invitrogen) in the same manner, which specifically binds to RNA. The labelled chondrocytes were transferred to a BioCoat Collagen I Culture Slide (Corning). After 4 h, the labelled chondrocytes adhered to the slide, and the labelled RAW264.7 cells were added. Fluorescent dye transfer was monitored over time with BZ-X800 and BZ-X800 analysis applications (KEYENCE Corp.).

To examine intercellular communication mediated by EVs, we tracked the RNA and plasma membrane components simultaneously. As described previously, we double-labelled RAW264.7 cells with 5 µM SYTO RNASelect and 25 µM CM-DiI. Following the unlabelled chondrocytes were allowed to adhere for 4 h, double-labelled RAW264.7 cells were added and cocultured for 4 h and 24 h. The samples were fixed with 4% paraformaldehyde for 15 min at 4 °C, mounted with mounting medium containing DAPI and analysed with BZ-X800 and BZ-X800 analysis applications (KEYENCE Corp.).

To examine the mode of communication between chondrocytes and RAW264.7 cells in more detail, we established 3 in vitro coculture groups: (1) direct coculture, (2) indirect coculture with an insert, and (3) direct coculture with an EV inhibitor. Each group included 3 lots of chondrocytes. Group (1) was tested as described above. For group (2), we first seeded unlabelled chondrocytes on Collagen I coverslips (Corning). Following adherence for 4 h, we placed a 12 mm Snapwell Insert with a 0.4 µm pore polyester membrane (Corning) on the coverslip, seeded double-labelled RAW264.7 cells onto the inner side of the insert, and cocultured the cells with the membrane. After 24 h of coculture, we removed the insert along with the seeded RAW264.7 cells. For group (3), we prepared a culture slide with unlabelled chondrocytes that had adhered as previously described. We also prepared double-labelled RAW264.7 cells, and after 2 h of preincubation with EV inhibitors [GW4869 (D1692-5MG, Sigma) or Y27632 (030-24021, Fujifilm Wako)], the cells were seeded on a culture slide with inhibitors (20 μM each). The cells were cocultured for 24 h, fixed with 4% paraformaldehyde for 15 min at 4 °C, mounted using mounting medium with DAPI, and analysed with BZ-X800 and BZ-X800 analysis applications (KEYENCE Corp.). For statistical analysis of intercellular communication, we acquired three fields of view with the same parameters for each group and counted the SYTO RNASelect-positive points within chondrocytes.

### Statistical analysis

Statistically significant differences between multiple groups were determined using one-way ANOVA, followed by Tukey’s test. A value of p < 0.05 was considered to be statistically significant. Normality was checked by Shapiro–Wilk test. All statistical analyses were performed with EZR (Saitama Medical Centre, Jichi Medical University, Saitama, Japan), which is a graphical user interface for R (The R Foundation for Statistical Computing, Vienna, Austria). More precisely, EZR is a modified version of the R commander designed to add statistical functions that are frequently used in biostatistics.

## Supplementary Information

Supplementary Figure 1.

## Data Availability

No datasets were generated or analysed during the current study.
