# Author Correction: Requirement of direct contact between chondrocytes and macrophages for the maturation of regenerative cartilage

**DOI:** 10.1038/s41598-021-03460-z

**Published:** 2022-01-13

**Authors:** Kengo Kanda, Yukiyo Asawa, Ryoko Inaki, Yuko Fujihara, Kazuto Hoshi, Atsuhiko Hikita

**Affiliations:** 1 Department of Sensory and Motor System Medicine, Graduate School of Medicine, The University of Tokyo, Tokyo, Japan; 2 Department of Tissue Engineering, The University of Tokyo Hospital, Tokyo, Japan; 3 Department of Tissue Stem Cell and Dental Life Science, Graduate School of Medicine, The University of Tokyo, Tokyo, Japan; 4 Department of Oral-Maxillofacial Surgery, Dentistry and Orthodontics, The University of Tokyo Hospital, Tokyo, Japan

Correction to: *Scientific Reports* https://doi.org/10.1038/s41598-021-01437-6, published online 18 November 2021

The original version of this Article contained an error in the legend of Figure 2.

“(**a–d**) Histological images of the outer bottom group at 1 week posttransplantation. Asterisks indicate the transplanted chondrocyte layer. (**a**) HE staining. (**b**) An enlarged view of the area indicated by the black rectangle in Fig. 1a. Capillaries are indicated by red arrowheads with enlarged views at the corners. (**c,d**) An immunofluorescent image of the same sample as in Fig. 1a. Asterisks indicate the transplanted chondrocyte layer. Alexa 594: F4/80, DAPI: nucleus. (**d**) An enlarged view of the area indicated by the yellow rectangle in (**c**). F4/80-positive cells (yellow triangle) were observed among the transplanted chondrocytes. (**e**–**g**) Immunofluorescent images of the outer bottom group sample at 2 weeks posttransplantation. Alexa 594: F4/80, DAPI: nucleus. Asterisks indicate the transplanted chondrocyte layer, and white circles indicate the host cell layer. (**f**,**g**) Enlarged views of areas indicated by yellow rectangles in (**d**).”

now reads:

“ (**a–d**) Histological images of the outer bottom group at 1 week posttransplantation. Asterisks indicate the transplanted chondrocyte layer. (**a**) HE staining. (**b**) An enlarged view of the area indicated by the black rectangle in (**a**). Capillaries are indicated by red arrowheads with enlarged views at the corners. (**c**,**d**) An immunofluorescent image of the same sample as in (**a**). Asterisks indicate the transplanted chondrocyte layer. Alexa 594: F4/80, DAPI: nucleus. (**d**) An enlarged view of the area indicated by the yellow rectangle in (**c**). F4/80-positive cells (yellow triangle) were observed among the transplanted chondrocytes. (**e**–**g**) Immunofluorescent images of the outer bottom group sample at 2 weeks posttransplantation. Alexa 594: F4/80, DAPI: nucleus. Asterisks indicate the transplanted chondrocyte layer, and white circles indicate the host cell layer. (**f**,**g**) Enlarged views of areas indicated by yellow rectangles in (**e**).”

The original Article has been corrected.

