# Supplementary material for: Requirement of direct contact between chondrocytes and macrophages for the maturation of regenerative cartilage
Source: Sci Rep. 2021 Nov 18;11:22476. doi: 10.1038/s41598-021-01437-6 (PMC8602279; doi:10.1038/s41598-021-01437-6)
Supplement: Supplementary file 1 — Supplementary Figure 1. [file 41598_2021_1437_MOESM1_ESM.pdf]

Supplementary Figure 1

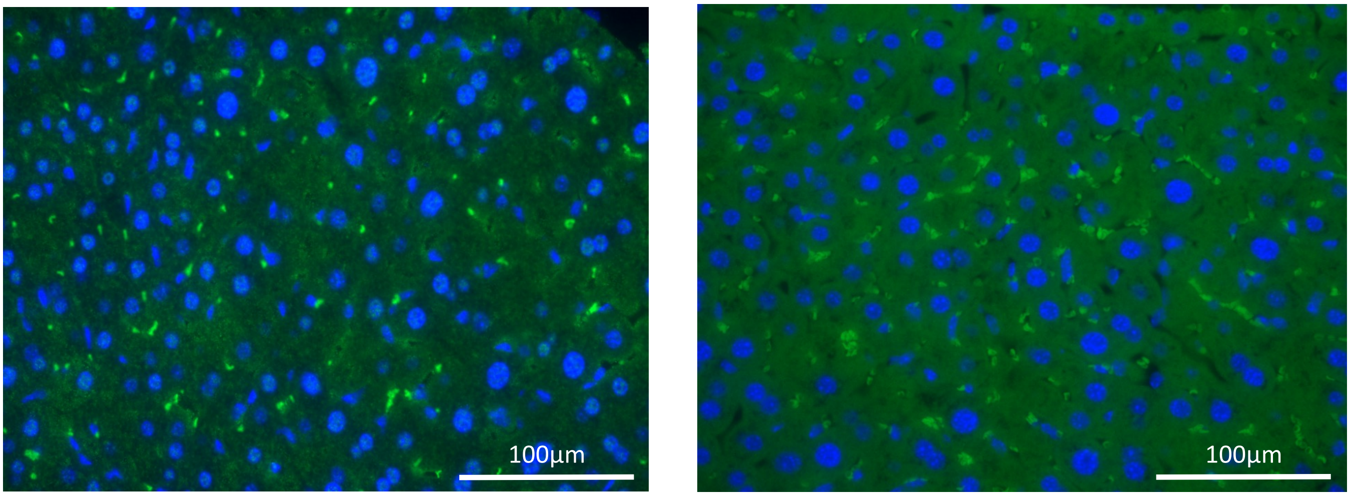

(a) iNOS (b) Arginase I

Immunofluorescence images of mouse liver.
(a) Alexa 488: iNOS, DAPI: nucleus.
(b) Alexa 488: Arginase I, DAPI: nucleus.
